# Functional indicators of response mechanisms to nitrogen deposition, ozone, and their interaction in two Mediterranean tree species

**DOI:** 10.1371/journal.pone.0185836

**Published:** 2017-10-03

**Authors:** Lina Fusaro, Adriano Palma, Elisabetta Salvatori, Adriana Basile, Viviana Maresca, Elham Asadi Karam, Fausto Manes

**Affiliations:** 1 Sapienza University of Rome, Department of Environmental Biology, Rome, Italy; 2 University of Naples “Federico II”, Biology Department, Naples, Italy; 3 Shahid Bahonar University of Kerman, Biology Department, Kerman, Iran; University of Naples Federico II, ITALY

## Abstract

The effects of nitrogen (N) deposition, tropospheric ozone (O_3_) and their interaction were investigated in two Mediterranean tree species, *Fraxinus ornus* L. (deciduous) and *Quercus ilex* L. (evergreen), having different leaf habits and resource use strategies. An experiment was conducted under controlled condition to analyse how nitrogen deposition affects the ecophysiological and biochemical traits, and to explore how the nitrogen-induced changes influence the response to O_3_. For both factors we selected realistic exposures (20 kg N ha^-1^ yr^-1^ and 80 ppb h for nitrogen and O_3_, respectively), in order to elucidate the mechanisms implemented by the plants. Nitrogen addition resulted in higher nitrogen concentration at the leaf level in *F*. *ornus*, whereas a slight increase was detected in *Q*. *ilex*. Nitrogen enhanced the maximum rate of assimilation and ribulose 1,5-bisphosphate regeneration in both species, whereas it influenced the light harvesting complex only in the deciduous *F*. *ornus* that was also affected by O_3_ (reduced assimilation rate and accelerated senescence-related processes). Conversely, *Q*. *ilex* developed an avoidance mechanism to cope with O_3_, confirming a substantial O_3_ tolerance of this species. Nitrogen seemed to ameliorate the harmful effects of O_3_ in *F*. *ornus*: the hypothesized mechanism of action involved the production of nitrogen oxide as the first antioxidant barrier, followed by enzymatic antioxidant response. In *Q*. *ilex*, the interaction was not detected on gas exchange and photosystem functionality; however, in this species, nitrogen might stimulate an alternative antioxidant response such as the emission of volatile organic compounds. Antioxidant enzyme activity was lower in plants treated with both O_3_ and nitrogen even though reactive oxygen species production did not differ between the treatments.

## Introduction

Mediterranean forests are subjected to challenging environmental conditions in the current global change context, since many stressors, individually or in combination, affect plant functionality simultaneously or successively over time [1]. Of more recent concern are the combined effects of ozone (O_3_) and nitrogen (N) on vegetation [2]. Monitoring activities in European countries have indicated that studies on O_3_ exposure effects are essential in Mediterranean regions [3], where the concurrence of high temperature and radiation promote the photo-stationary cycle toward high O_3_ concentration during the late spring and summer seasons [4].

O_3_ impacts forests by increasing the oxidation load, thereby triggering the production of reactive oxygen species (ROS) that lead to alterations of functional processes at different levels [5,6]. The production of ROS activates the detoxifying barrier in the apoplast and enzymatic activity at the symplastic level that have high metabolic cost [7,8], and the capacity to increase antioxidant defences is recognized as a key factor in determining O_3_ tolerance [9–12].

Leaf gas exchange is also affected by O_3_ through a direct impact on stomatal guard cell functionality [13,14] or stomatal number [15], as well as owing to a decrease in the photochemical and carboxylation efficiency [15–18]. Leaf structural traits such as leaf mass area (LMA), in addition to leaf nitrogen and carbon concentrations, have been found to reveal ozone sensitivity and tolerance in different species [19,20], where species with low LMA and high leaf nitrogen concentration show higher O_3_ sensitivity [11,21]. Further, O_3_ can adversely influence these functional traits, accelerating leaf senescence processes [22–24].

Nitrogen deposition represents an additional threat for Mediterranean forests adapted to low nitrogen availability [25]. During the last decades many studies have evaluated the effects of nitrogen on plant biodiversity and carbon balance or assimilation capacity [26–30], but many of them have been conducted on pastures [31], boreal and temperate forest species [26], or in Chaparral species [32,33]. The implications concerning Mediterranean forests are still scarce [34], and knowledge regarding the response of the large plethora of functional traits to increasing nitrogen deposition for forest species is lacking [15,35,36]. In Italy, the average nitrogen throughfall, in terms of NO_3_NH_4_, measured using the network of permanent monitoring stations, ranges between 4 and 29 kg N ha^-1^ yr^-1^ [37], and the critical loads indicated for Mediterranean forest ecosystems that fall within the range of 10 to 15 kg N ha^-1^ yr^-1^ have low reliability owing to the lack of experimental evidence [26].

Previous studies have shown that higher nitrogen availability can increase stomatal conductance [38,39], entailing a potential harmful increase in O_3_ uptake. However the effects of nitrogen on hydraulic architecture and stomatal conductance are still contradictory [40]. Higher leaf nitrogen contents can result in photosynthetic enhancements owing to the key role that nitrogen has in Calvin cycle and proteins [41], or by the increase of leaf area. Recent studies on the interaction between O_3_ and nitrogen deposition in deciduous species have highlighted that O_3_ reduced the nitrogen availability for photosynthesis in *Fagus crenata* [42]; further, the positive effect on root development owing to nitrogen, is lost at higher O_3_ levels in *Quercus robur* L. [43]. An antagonistic effect was detected on root starch concentrations, where higher nitrogen levels alleviated the negative impact of ozone [39]. Recent findings suggested that the interactions between O_3_ and nitrogen depend on the concentration of these two factors and can change throughout the growing season [2]. Therefore, experiments under controlled conditions are required to better elucidate the mechanisms underlying the influence of nitrogen on the key functional traits that are involved in pollutant uptake or antioxidant defence mechanisms [2].

Moreover, species can remarkably differ in nitrogen absorption depending on the successional stage and resource allocation strategy [44,45]. Deciduous species tend to allocate nitrogen to ribulose-1, 5-bisphosphate carboxylase/oxygenase (Rubisco), or to light-harvesting components in order to enhance the photosynthetic capacity; in contrast, in evergreen species, nitrogen is preferentially allocated to the cell walls, leading to an increase in the persistence of leaves [46,47], as well as toughness and chemical defence [48].

In this framework, we performed an experiment under controlled conditions to investigate how *Fraxinus ornus* L. and *Quercus ilex* L. react to nitrogen addition, and how nitrogen availability can influence the response mechanisms to O_3_. For both factors, we selected realistic exposures (20 kg N ha^-1^ yr^-1^ and 80 ppb h for N and O_3_, respectively), since an acute exposure could hinder the elucidation of mechanisms implemented by the plants [2]. We focused on these two species that typically co-occur in Mediterranean forests [49], and have different functional traits and successional positions. *F*. *ornus* is typical of early successional stages, with a rapid growth strategy, whereas *Q*. *ilex* belongs to the mature stage of a succession with a slow growth strategy and a conservative patterns of nutrient use [50]. Moreover, previous studies have suggested that *F*. *ornus* is moderately sensitive to O_3_ [51], whereas *Q*. *ilex* was considered to be tolerant to this pollutant [11,15,52]; however, to the best of our knowledge, these two species have not yet been compared directly.

Owing to leaf habit and more flexible patterns of nitrogen uptake in *F*. *ornus* than in *Q*. *ilex* [53], we hypothesized that *F*. *ornus* uptakes a large amount of nitrogen and allocates higher fraction to photosynthetic tissues; in contrast, in *Q*. *ilex*, we expected that nitrogen would be allocated to non-photosynthetic compounds. Since both the hypothesized species-specific responses to nitrogen are related to O_3_ tolerance, nitrogen addition could lead to mitigation of O_3_ detrimental effects on functional traits. The present study provides new data on *F*. *ornus* and *Q*. *ilex* that would be useful to improve the risk assessment for Mediterranean forests subjected to nitrogen deposition and ozone. Furthermore, understanding the mechanisms underlying functional trait shifts under multistress environments could facilitate the forecasting of forests’ responses to global change and addressing biodiversity conservation in the future.

## Materials and methods

### Growth conditions

Two-year-old seedlings of *F*. *ornus* and *Q*. *ilex*, obtained from the nursery of Aurunci Regional Park (Central Italy), were transported to the experimental garden of the Department of Environmental Biology, Sapienza University of Rome on 18 May 2016. Plants were transferred to 7L pots along with their clods and the remaining pot volume was filled with a mixture of sand, turf, and perlite. The experiment was conducted in a ‘walk-in’ chamber facility, consisting of two closed chambers (2.5 m × 3.9 m × 3 m h): one was used as control and one for O_3_ fumigation [54]. Air temperature was maintained at 27.9 ± 1.8°C during the day and at 22.7 ± 0.9°C at night. The relative humidity was 61 ± 6.1%.

In each chamber, a photosynthetic active radiation of approximately 700 μmol m^−2^ s^−1^ was provided for 12 h per day by using 6 metal halide lamps (1000 W; Philips HPI-T). The microclimatic conditions were monitored at 5-min intervals and did not differ significantly between the chambers. In each chamber, plants were randomly relocated daily to reduce possible position effects. During the entire experimental period, all plants were watered in order to maintain soil close to field capacity and avoid water stress.

### Experimental design

After the plants were acclimated for 30 days to the chamber environmental conditions, 20 plants per species were randomly divided into four experimental sets (C, N, O_3_, and O_3_N). Ten plants per species were assigned to the control chamber and thus randomly divided as follows: five plants to the control experimental set (C), and five plants to nitrogen addition experimental set (N). Ten plants per species were assigned to the fumigated chamber and thus randomly divided as follows: five plants to the O_3_ treatment (O_3_), and five plants to the interaction experimental set, treated with both N and O_3_ (O_3_N).

The fertilizer was divided into 7 aliquots and applied throughout the experimental period as an aqueous solution. For this, 100 mL of deionised water was weekly added to each pot with different doses of ammonium nitrate (NH_4_NO_3_): 0 mg for C plants and 0.031 mg for N treatment. The final nitrogen dose was equal to 20 kg N ha^-1^ yr^-1^ based on the soil surface area. The ozone fumigation was started after five nitrogen additions, when the cumulative dose was roughly equivalent to 14 kg N ha^-1^ yr^-1^, which falls in the upper limit of the threshold load currently indicated as critical for Mediterranean vegetation. The acclimation to nitrogen addition phase and fumigation period lasted 30 days and 10 days, respectively.

During the fumigation period, the C and N experimental sets were kept in the control chamber under filtered air (O_3_ = 0 to maximal 5.8 ppb). The O_3_ and O_3_N sets were placed in the fumigation chamber and exposed for 10 consecutive days to a mean hourly O_3_ concentration of 87.00 ± 0.5 ppb for 5 h per day simulating a concentration found in the Mediterranean rural area during the summer period [55,56]. The cumulative exposure was 2585.47 ppb h, expressed as AOT40, calculated by summing up all of the exceedances of the hourly O_3_ concentration above 40 ppb during the daylight hours [56]. O_3_ was generated in the fumigation chamber by flowing pure oxygen on a UV light source (Helios Italquartz, Milan, Italy), and then added to the chamber air inlet via a Teflon tube. The O_3_ concentration at plant height was continuously monitored using a photometric O_3_ detector (Model 205;, 2B Technologies, Boulder, CO, USA).

Leaf gas exchange and chlorophyll (Chl) *a* fluorescence were measured every three days, particularly on the first, fourth, seventh and tenth day of fumigation (DOF). Immediately after the end of fumigation, leaves for biochemical analysis (antioxidant activity) and structural measurements (nitrogen and carbon concentration, leaf mass area) were sampled; P_N_/C_i_ curves were performed within two days. Further details about the measurements are provided below.

### Gas exchange measurements

Steady state measurements of gas exchange were performed using a portable infrared gas analyser (CIRAS-2; PP-System International, Amesbury, MA). The net photosynthesis (P_N_, μmolCO_2_ m^-2^ s^-1^), leaf transpiration (E, mmolH_2_O m^-2^ s^-1^), stomatal conductance (gs, mmolH_2_O m^-2^ s^-1^) and sub-stomatal CO_2_ concentration (C_i_, ppm) were simultaneously measured. The instantaneous water use efficiency (WUE, μmol CO_2_ mmol H_2_O^-1^) was calculated as the ratio between net photosynthetic rates and transpiration rates, and the ratio of substomatal and ambient CO_2_ concentration, C_i_/C_a_, was determined. All measurements were performed using fully developed leaves.

### *Chl a* fluorescence measurements and application of the JIP-test

The Chl *a* fluorescence was measured using a Handy PEA direct fluorometer (Hansatech Instruments, Norfolk, UK) on the same days, hours, and leaves as those used for steady state gas exchange measurements. After a dark adaptation period of 40 min, obtained using specific leaf clips, the measured leaves were exposed to a saturating red light pulse (peak 650 nm) of 3000 μmol photons m^−2^ s^−1^, for 1 s, thereby generating a fluorescence transient (FT). The FT, plotted on a logarithmic timescale, showed a polyphasic behaviour, the different steps of which corresponded to a specific stage in the electron chain between reaction centres of photosystem II (PSII) and end acceptors of photosystem I (PSI) [57,58]. The first part of the transient curve (O–J) is called ‘single turnover region’. It expresses the photochemical events, providing information regarding the reduction of plastoquinone. The J–I–P region of the FT is called ‘multiple turnover region’ and reflects the velocity of ferredoxin reduction beyond PSI. In particular, the I–P region reflects the velocity and quantity of ferredoxin and NADP reduction via electron donation of PSI. The JIP-test was applied to the FT, and the following parameters were calculated from each curve:

- φ_Po_: maximum quantum yield of primary photochemistry expresses the probability that an absorbed photon will be trapped by the PSII reaction centre;
- J-phase: expression of the efficiency with which a trapped exciton can move an electron into the electron transport chain from plastoquinone to the intersystem electron acceptors;
- IP-phase: expression of the efficiency of electron transport around PSI to reduce the final acceptors of the electron transport chain, i.e. ferredoxin and NADP^+^
- PI_tot_: a multiparametric expression that synthesizes the potential for energy conservation from photons absorbed by PSII to the reduction of PSI end acceptors.

### *P*_*N*_*/C*_*i*_ response curves

The response of net photosynthesis to the variation of substomatal CO_2_ concentration was measured on the same leaves used for steady state gas exchange. Two intercalibrated CIRAS2 were used for simultaneous measurements in *F*. *ornus* and *Q*. *ilex*. The P_N_/C_i_ curves were constructed following Long and Bernacchi, 2003 [59]. Cuvette environment was maintained at 60% relative humidity and 25°C; photosynthetic active radiation was mantained at the saturating value of 1000 μmol m^-2^s^-1^. The assimilation rate under CO_2_ saturation (P_Nmax_) was measured and the maximum electron transport rate driving regeneration of ribulose 1,5-bisphosphate (J_max_, mol m^-2^ s^-1^) was calculated according to Loustau et al. 1999 [60]. The CO_2_ compensation point Γ (ppm) was derived and the *in vivo* apparent Rubisco activity (V_cmax_, mol m^-2^s^-1^) was calculated as the angular coefficient of the linear part of the curve. Data at very low [CO_2_], which can be limited by Rubisco deactivation, were excluded from the analysis [61].

### Leaf chemistry and derivation of the photosynthetic nitrogen use efficiency

The total leaf nitrogen and carbon concentrations (N_L_, C_L_, % dry mass) were determined using the Dumas micro-combustion technique (Eurovector EA 3000; Milan, Italy) on the same dried leaf samples used for the calculation of sclerophylly degree (see paragraph Leaf structural and total biomass traits). Samples were ground in liquid nitrogen, and five subsamples were weighed using a precision balance (MJ-300; d = 0.001g) before the analysis. photosynthetic nitrogen-use efficiency (PNUE) was calculated as the ratio of instantaneous P_Nmax_ to nitrogen on an area basis.

### Antioxidant enzymes

Antioxidant enzyme activities were determined using fresh leaf material, which was extracted as described previously [62]. All reagents for oxidative stress detection were purchased from Sigma-Aldrich (St. Louis, MO, USA). ROS production was detected using the general oxidative stress cell-permeant 2′,7′dichlorodihydrofluorescein diacetate dye. This dye passively diffuses into the cells and interacts with endogenous esterases, which cleave the diacetate groups. The stock solution of the dye (25 μM in DMSO) was diluted to a final concentration of 5 μM. Fluorescence was monitored using a fluorescence spectrophotometer, with an excitation wavelength of 350 nm and an emission wavelength of 600 nm. The increase in fluorescence intensity yielded the ROS quantity.

The superoxide dismutase (SOD) activity was determined using an SOD assay kit WST (Sigma–Aldrich) according to manufacturer’s instructions. The SOD activity (inhibition of activity) was calculated by measuring the decrease in the colour development at 440 nm. Catalase (CAT, EC 1.11.1.6) activity was measured using a commercial CAT assay kit (Sigma–Aldrich) following manufacturer’s protocol. CAT activities were calculated and expressed as a decrease in absorbance at 240 nm due to H_2_O_2_ consumption. The total ascorbate peroxidase (APX, EC 1.11.1.11) activity of leaves was assayed by monitoring the decrease in absorbance at 290 nm due to ascorbate oxidation [63]. The concentration of ascorbic acid (ASC) was measured as described by [64]. Briefly, total ascorbate was determined after the reduction of oxidised ascorbic acid (DHA) to ASC with 1,4-dithiothreitol, and the concentration of DHA was estimated from the difference between the total ascorbate pool (ASA plus DHA) and ASC. Glutathione (GSH) content was determined at 412 nm by using 5,5ʹ-dithiobis(2-nitrobenzoic acid), according to the spectrophotometric method of [65].

### Leaf structural traits

The degree of sclerophylly was estimated by assessing the leaf mass area (LMA, g cm^-2^). After petiole exclusion, the leaf area was measured using Image Lab software (http://en.freedownloadman-ager.org/Windows-PC/Image-Lab.html), and after the samples were dried at 80°C to constant weight, the leaf dry weight (g) was measured.

### Statistical analysis

The effect of time on ecophysiological measurements (gas exchange and Chl *a* fluorescence) was analysed using repeated measurement ANOVA with nitrogen and O_3_ treatments as between-subjects factors. Two-way ANOVA, with nitrogen and O_3_ as fixed factors, with their interaction factor, was used to analyse the ecophysiological measurements during each sampling date (DOF1, DOF 4, DOF 7, and DOF 10), and to test the differences between treatments on the P_N_/C_i_ curve parameters and on the biochemical and structural measurements performed at the end of the experiment. Two-way ANOVA was followed by post hoc Student–Neuman–Keuls test at *p* < 0.05 when necessary. All analyses were performed using Statistica software, version 7.0 (StatSoft, Tulsa OK, USA).

## Results

### Steady-state gas exchange

Advancement of time (e.g plant developmental stage) affected all the gas exchanges parameters for both species, with the exception of WUE in *F*. *ornus*. In this species, a significant time × nitrogen interaction was noted (Table 1A) for all parameters. The time × O_3_ interaction was significant for WUE and C_i_/C_a_ in *Q*. *ilex*, whereas the three-level interaction was significant for WUE and C_i_/C_a_ in both species and for g_s_ only in *Q*. *ilex*.

**Table 1:** Analysis of variance of the gas exchange parameters for *F*. *ornus* and *Q*. *ilex*.

*F*. *ornus*					*Q*. *ilex*			
Factors	P_N_	g_s_	WUE	C_i_/C_a_	P_N_	g_s_	WUE	C_i_/C_a_
**a) *Repeated measures ANOVA***								
Time	**0.000**	**0.003**	0.077	**0.023**	**0.001**	**0.000**	**0.000**	**0.000**
Time × N	0.058	**0.004**	**0.000**	**0.000**	0.612	0.752	0.594	0.559
Time × O_3_	0.435	0.589	0.173	0.069	0.606	0.070	**0.017**	**0.047**
Time × N × O_3_	0.521	0.109	0.052	**0.025**	0.124	**0.010**	**0.005**	**0.006**
**b) *Two-way ANOVA***								
**DOF 1**								
N	**0.033**	**0.002**	**0.000**	**0.000**	0.992	0.867	0.715	0.813
O_3_	0.212	0.055	0.672	0.936	0.076	0.177	0.678	0.861
N × O_3_	**0.013**	**0.008**	**0.004**	**0.005**	0.509	**0.050**	**0.036**	0.055
**DOF 4**								
N	**0.018**	0.071	0.640	0.370	0.256	0.299	0.335	0.355
O_3_	**0.001**	0.051	0.885	0.900	**0.009**	**0.006**	**0.025**	**0.032**
N × O_3_	0.050	0.202	0.970	0.670	0.151	0.469	0.505	0.560
**DOF 7**								
N	0.969	0.922	0.901	0.540	0.074	0.160	0.995	0.706
O_3_	0.058	**0.035**	**0.022**	**0.001**	**0.029**	**0.004**	**0.026**	**0.012**
N × O_3_	0.389	0.728	0.927	0.950	1.000	0.915	0.790	0.754
**DOF 10**								
N	0.432	0.725	0.185	0.162	0.146	0.117	0.496	0.658
O_3_	**0.013**	**0.014**	0.230	0.354	**0.006**	**0.000**	**0.001**	**0.004**
N × O_3_	**0.008**	0.050	0.748	0.944	0.597	0.316	0.858	0.757

Analysis of variance on the steady-state gas exchange parameters: Repeated measures ANOVA (a) and two-way ANOVA (b) of time, nitrogen, O_3_, and their interaction effects are reported for each measurement date. DOF = day of fumigation. Significant *p*-value (*p* < 0.05) are marked in bold; ‘quasi’ significant values (0.1 > *p* > 0.05) are underlined.

**Fig 1:**
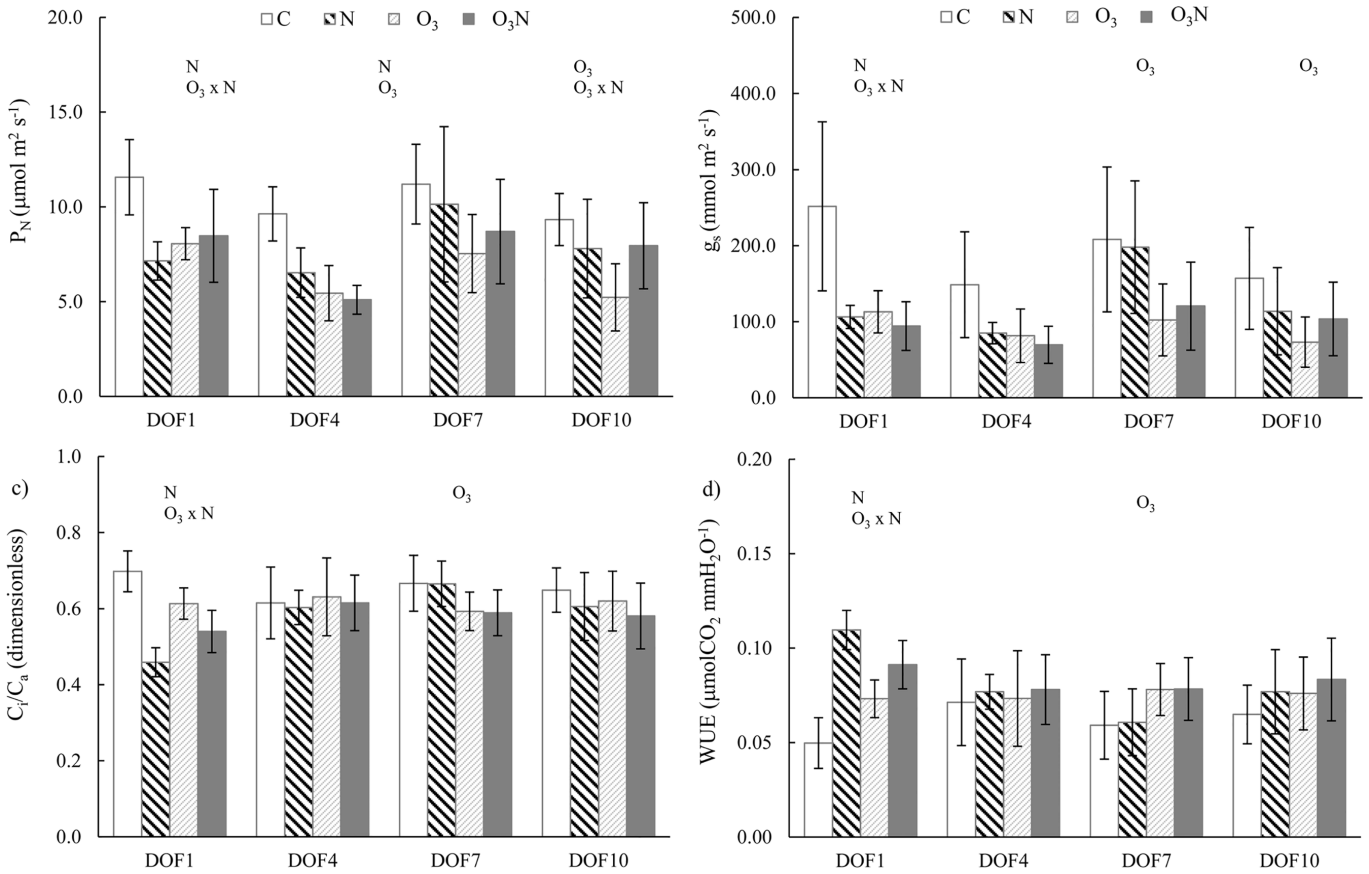
Trend of steady-state gas exchange parameters in *F*. *ornus*. The trend is shown as the mean and standard deviation (n = 5) for each treatment. Measurements were performed at the first, fourth, seventh, and tenth day of fumigation (DOF). Symbols over the bars indicate the significant factors (*p* < 0.05) affecting the gas exchange parameters: N, nitrogen effect; O_3_, ozone effect; O_3_ × N, interaction.

In particular, comparison of the gas exchange parameters of *F*. *ornus* for each sampling date (Fig 1) revealed that nitrogen affected all the assayed parameters at DOF 1, with a decrease in P_N_, g_s_, and C_i_/Ca and an increase in WUE. However, at DOF4, only P_N_ was affected and, at the following sampling dates, no difference from C values was found.

O_3_ began to affect P_N_ and g_s_ (-75% and -82% compared to those in the control, respectively) on DOF 4, lasting with the same order of magnitude through DOF 7 to 10. The interaction significantly affected P_N_ and g_s_ for low or high O_3_ exposure (DOF 1 and 10, AOT40 254.22 and 2585.47 ppb h, respectively); the direction of the interaction remained the same, where the decrease of P_N_ and g_s_ relative to the control was less pronounced in O_3_N plants than in O_3_ alone.

**Fig 2:**
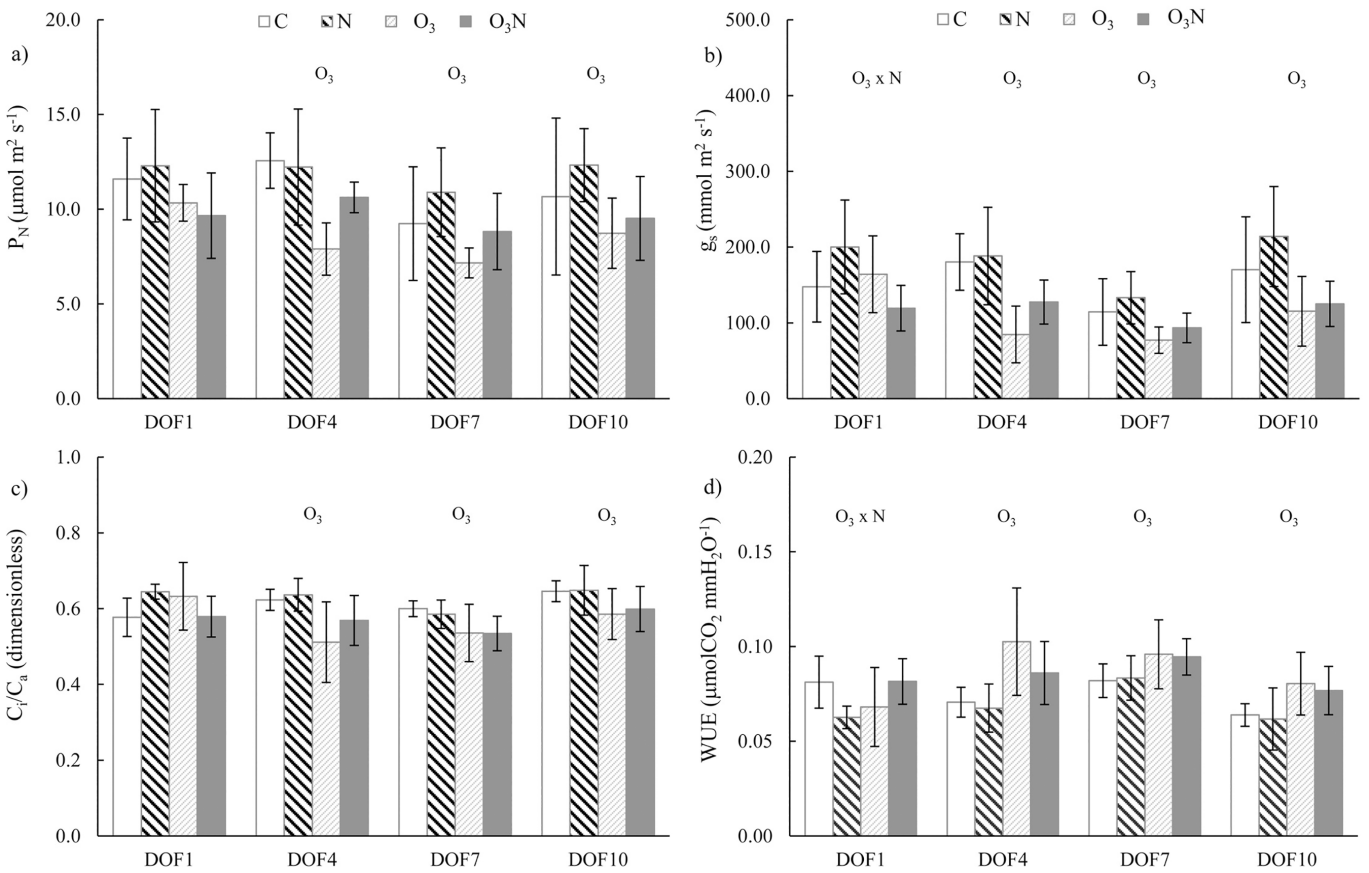
Trend of steady-state gas exchange parameters in *Q*. *ilex*. The trend is shown as the mean and standard deviation (n = 5) for each treatment. Measurements were performed at the first, fourth, seventh, and tenth day of fumigation (DOF). Symbols over the bars indicate the significant factors (*p* < 0.05) affecting the gas exchange parameters: N, nitrogen effect; O_3_, ozone effect; O_3_ × N, interaction.

For *Q*. *ilex* nitrogen led to the increase in both P_N_ and g_s_ relative to those in C (from +5% to 15% depending on DOF); however, the variability in the data led to *p* > 0.05 at each DOF (Fig 2). The main factor affecting gas exchange was O_3_, entailing a decrement of P_N_ (-60% at DOF 4, - 28% at DOF 7 and -23% at DOF10) because of stomatal limitation as indicated by C_i_/C_a_ reduction. The interaction was present on DOF 1, for low O_3_ exposure (AOT40 254.22 ppb h) only for g_s_, where the reduction in O_3_N experimental set was higher than in the O_3_ set. At DOF 4 and DOF 10, the reduction of P_N_ and g_s_ in O_3_N was less pronounced than that in O_3_, even if the interaction effect was not significant (Table 1B).

### *Chl a* fluorescence measurements

The repeated measures ANOVA showed that the photosystems functionality was not affected by time in both species (Table 2A).

**Table 2:** Analysis of variance of JIP-test parameters for *F*. *ornus* and *Q*. *ilex*.

*F*. *ornus*					*Q*. *ilex*			
Factors	φ_P0_	J-phase	IP-phase	PI_tot_	φ_P0_	J-phase	IP-phase	PI_tot_
**a) *Repeated measures ANOVA***								
Time	0.576	0.781	0.082	0.141	0.249	0.369	0.363	0.419
Time × N	0.670	0.959	0.359	0.250	0.739	0.753	0.565	0.286
Time × O_3_	0.853	0.979	0.832	0.478	0.879	0.604	0.662	0.521
Time × N × O_3_	0.062	0.588	0.092	0.220	0.184	0.204	0.788	0.670
**b) *Two-way ANOVA***								
**DOF 1**								
N	0.016	**0.000**	0.075	**0.000**	0.788	0.213	**0.044**	**0.044**
O_3_	0.625	**0.004**	0.726	**0.005**	0.118	0.077	**0.001**	**0.002**
N × O_3_	0.683	0.589	0.321	0.361	0.630	0.870	**0.016**	0.156
**DOF 4**								
N	**0.000**	**0.042**	**0.000**	**0.000**	0.987	0.991	0.973	0.816
O_3_	**0.000**	0.570	0.443	**0.1**	0.022	0.365	**0.002**	**0.001**
N × O_3_	0.552	0.435	**0.000**	**0.000**	0.521	0.313	0.789	0.776
**DOF 7**								
N	0.072	**0.025**	**0.056**	**0.028**	0.734	0.114	0.151	0.209
O_3_	0.467	0.781	0.267	0.187	0.056	**0.013**	**0.001**	**0.001**
N × O_3_	0.064	**0.014**	0.456	0.820	0.126	0.268	0.936	0.282
**DOF 10**								
N	**0.038**	0.765	**0.000**	**0.000**	0.744	0.374	0.834	0.178
O_3_	0.795	0.179	**0.016**	0.114	0.195	**0.053**	**0.003**	**0.000**
N × O_3_	0.239	0.723	**0.009**	**0.001**	0.436	0.642	0.424	0.397

Analysis of variance on JIP-test parameters: Repeated measures ANOVA (a) and two-way ANOVA (b) of time, nitrogen, O_3_, and their interaction effects, reported for each measurement date. DOF = day of fumigation. Significant *p*-value (*p* < 0.05) are marked in bold; ‘quasi’ significant values (0.1 > *p* > 0.05) are underlined.

**Fig 3:**
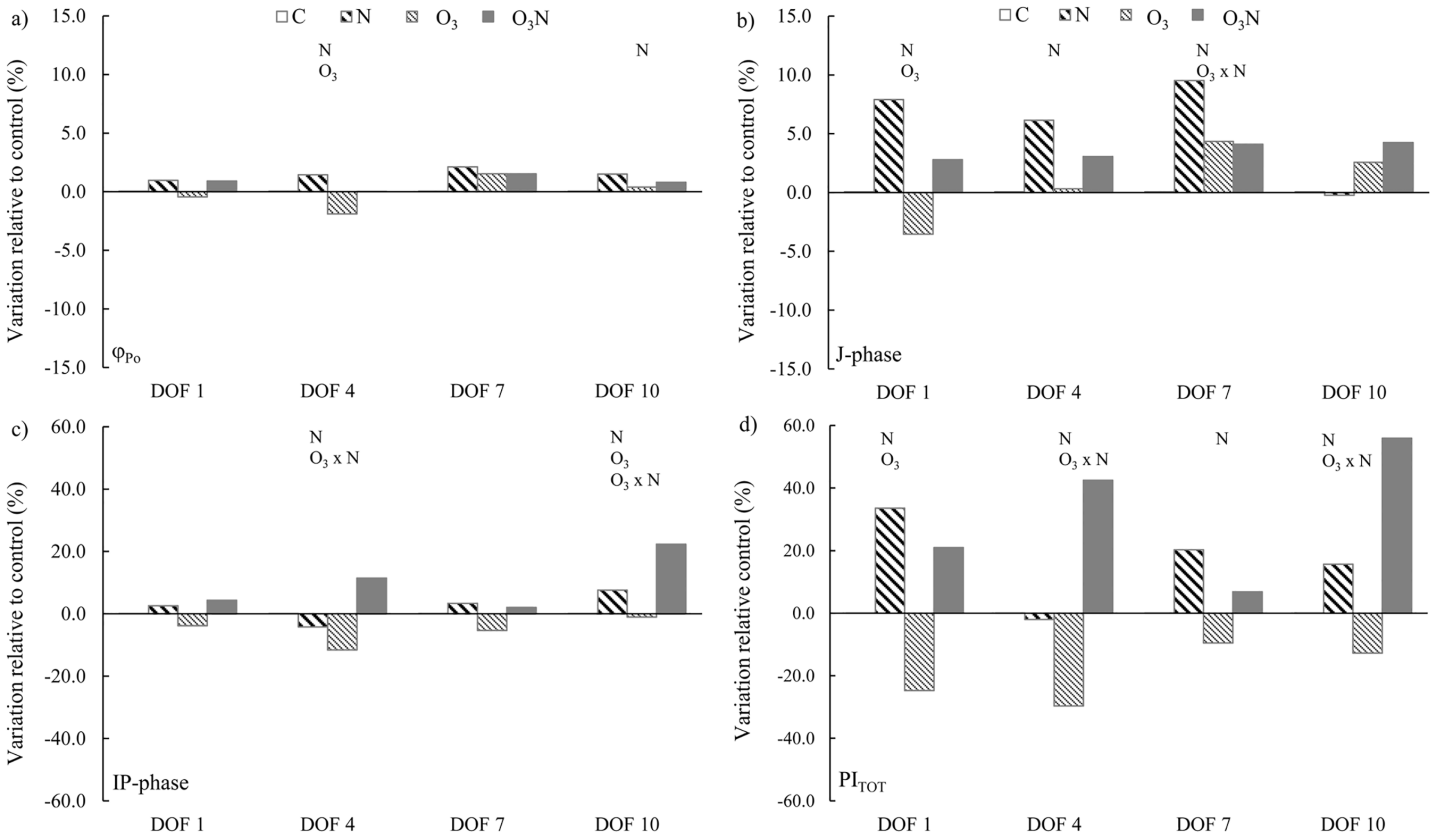
Trend of JIP-test parameters in *F*. *ornus*. The histograms indicate the percentage variation of each parameter of all treatments in relation to those in control plants. Measurements were performed at the first, fourth, seventh, and tenth day of fumigation (DOF). Symbols over the bars indicate the significant factors (*p* < 0.05) affecting the gas exchange parameters: N, nitrogen effect; O_3_, ozone effect; O_3_ × N, interaction.

In *F*. *ornus*, nitrogen enhanced the primary reactions characterizing the single turnover region of the fluorescence transient (Fig 3A and 3B). A slight, but significant increase of φ_Po_ occurred at DOF 4 and DOF 10, and the J-phase was affected from DOF 1 to DOF 7 (Table 2B). Nitrogen influenced the IP-phase (rate of reduction of end acceptors ferredoxin and NADP) on DOF 4 and 10 and the overall functionality of photosystems as showed by the trend of PI_tot_. O_3_ affected the JIP-test parameters since DOF 1, decreasing both IP-phase and PI_tot_ relative to those in the control. The interaction between nitrogen and O_3_ was evident on PI_tot_ (DOF 4, 10), with nitrogen ameliorating the detrimental effect of O_3_ photosystem functionality (Fig 3D).

**Fig 4:**
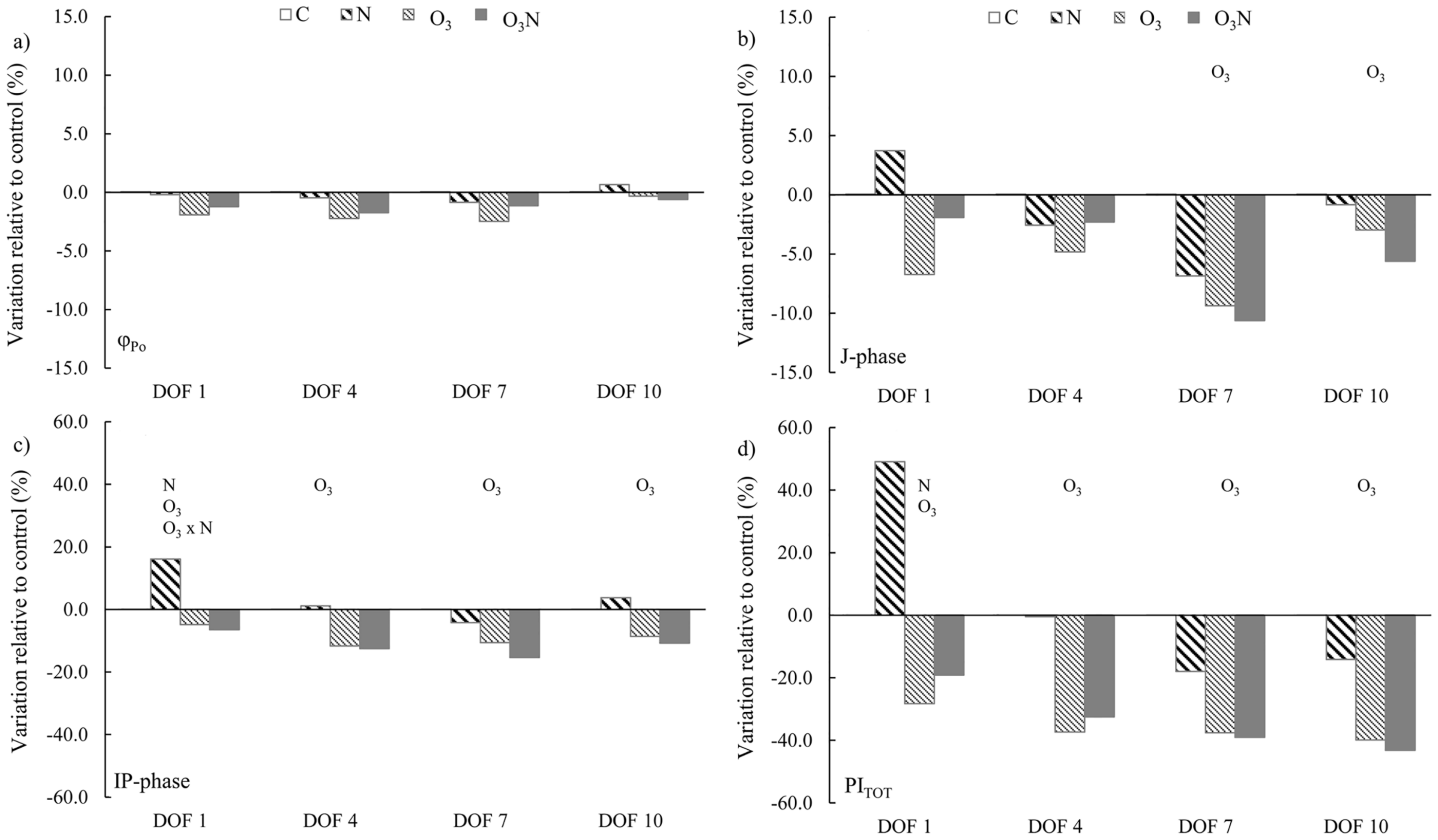
Trend of JIP-test parameters in *Q*. *ilex*. The histograms indicate the relative variation of each parameter of all treatments in relation to those in control plants. Measurements were performed at the first, fourth, seventh, and tenth day of fumigation (DOF). Symbols over the bars indicate the significant factors (*p* < 0.05) affecting the gas exchange parameters: N, nitrogen effect; O_3_, ozone effect; O_3_ × N, interaction.

In *Q*. *ilex*, the effect of nitrogen and interaction between factors on photosystems functionality was marginal (Fig 4; Table 2), affecting IP-phase and PI_tot_ on DOF1 only. The main driver of photosystem functionality was O_3_, which influenced the primary photochemistry (Fig 4B) only during the final phase of O_3_ exposure (DOF 7 and 10), whereas IP-phase and PI_tot_ (Fig 4C and 4D) were affected by O_3_ since DOF1.

### *P*_*N*_*/C*_*i*_ response curves, leaf chemistry, and structural traits

The parameters derived from P_N_/C_i_ curves, together with PNUE, chemical and structural leaf traits such as nitrogen and carbon concentration for the two species (N_L_; C_L_), their ratio, and LMA are shown in Table 3.

**Table 3:** Parameters derived from the P_N_/Ci response curves performed at the end of the experimental period and the chemical and structural leaf traits.

***F*. *ornus***					***p***		
**Parameters**	**C**	**N**	**O**_**3**_	**O**_**3**_**N**	**N**	**O**_**3**_	**O**_**3**_**N**
**P**_**Nmax**_	25.64 ± 4.40	32.23 ± 4.24	22.73 ± 0.19	22.14 ± 3.65	**0.015**	**0.01**	0.09
**V**_**cmax**_	0.078 ± 0.01	0.067 ± 0.01	0.051 ± 0.00	0.057 ± 0.01	0.742	**0.03**	0.23
**J**_**max**_	106.6 ± 18.44	136.5 ± 13.93	98.32 ± 6.26	91.58 ± 15.58	**0.012**	**0.01**	**0.04**
**Γ**	48.67 ± 3.68	56.2 ± 18.02	70.65 ± 23.13	52.20 ± 1.68	0.555	0.34	0.18
**PNUE**	1.9 ± 0.64	1.55 ± 0.28	1.40 ± 0.44	2.19 ± 1.07	0.59	0.86	0.19
**N**_**L**_	1.71 ± 0.09	2.14 ± 0.45	1.26 ± 0.1	1.56 ± 0.32	0.058	**0.01**	0.71
**C**_**L**_	44.73 ± 0.27	45.31 ± 0.6	44.25 ± 0.45	44.66 ± 0.42	0.094	0.06	0.76
**C**_**L**_**/N**_**L**_	26.15 ± 1.21	21.7 ± 3.97	35.21 ± 2.75	29.49 ± 6.66	0.067	**0.01**	0.8
**LMA**	0.013 ± 0.001	0.013 ± 0.001	0.011 ± 0.001	0.012±0.001	0.081	**0.003**	0.539
***Q*. *ilex***					***p***		
	**C**	**N**	**O**_**3**_	**O**_**3**_**N**	**N**	**O**_**3**_	**O**_**3**_**N**
**P**_**Nmax**_	24.73 ± 3.91	29.9 ± 1.50	23.54 ± 5.41	28.67 ± 3.10	**0.003**	0.370	0.989
**V**_**cmax**_	0.076 ± 0.01	0.086 ± 0.009	0.074 ± 0.014	0.10 ± 0.017	0.059	0.498	0.368
**J**_**max**_	105.9 ± 12.72	125.8 ± 4.19	102.3 ± 0.99	129.7 ± 8.64	**0.000**	0.982	0.438
**Γ**	67.69 ± 1.67	60.06 ± 13.14	67.11 ± 1.92	74.36 ± 12.98	0.972	0.238	0.204
**PNUE**	3.44 ± 0.97	3.00 ± 0.94	1.84 ± 0.13	2.2 ± 0.54	0.925	**0.021**	0.372
**N**_**L**_	1.26 ± 0.14	1.44 ± 0.35	1.14 ± 0.07	1.36 ± 0.23	0.158	0.479	0.888
**C**_**L**_	44.43 ± 0.48	45.24 ± 0.37	45.28 ± 0.21	45.55 ± 0.35	**0.036**	**0.026**	0.243
**C**_**L**_**/N**_**L**_	35.6 3± 4.03	32.57 ± 7.51	39.79 ± 2.36	34.01 ± 5.45	0.178	0.378	0.663
**LMA**	0.014 ± 0.001	0.013 ± 0.001	0.015 ± 0.001	0.012 ± 0.001	**0.000**	0.98	**0.01**

P_Nmax_, (μmol m^-2^ s^-1^) = maximum rate of net photosynthesis; V_cmax_, (mol m^-2^ s^-1^) = in vivo apparent Rubisco activity; J_max_, (μmol m^-2^ s^-1^) = maximum rate of electron transport; Γ, (ppm) = CO_2_ compensation point; PNUE, photosynthetic nitrogen use efficiency (μmol mol^-1^ s^-1^); N_L_, nitrogen at leaf level (%); C_L_, carbon concentration at leaf level (%); C_L_/N_L_, ratio between carbon and nitrogen at leaf level; Leaf Mass Area (LMA, g cm^-2^). Data are shown as the mean ± standard deviation (n = 5) for each treatment. On the right panel, results of two-way ANOVA for each parameter are shown. Significant *p*-value (*p* < 0.05) are marked in bold; quasi ‘significant’ values (0.1 > *p* > 0.05) are underlined.

In *F*. *ornus*, P_Nmax_ increased significantly after nitrogen addition (final dose, 20 kg N ha^-1^ yr^-1^), decreasing in response to O_3_ (Table 3). The nitrogen concentration at the leaf level increased in the N experimental set, although slightly significantly (*p* = 0.058). The main driver of photosynthesis was O_3_, leading to a reduction of P_Nmax_, V_cmax_ and J_max_ relative to those in C plants. The N_L_ decreased because of O_3_ fumigation, entailing a significant change in the C_L_/N_L_ ratio. The interaction between factors significantly affected J_max_: O_3_ limited the positive effect of nitrogen. In *F*. *ornus*, nitrogen did not affect the LMA, whereas this parameter was reduced by O_3_ (Table 3).

Interestingly, in *Q*. *ilex*, the response curves parameters were affected by nitrogen, thereby enhancing P_Nmax_ as well as the apparent maximum electron transport rate contributing to RuBP regeneration (J_max_). Nitrogen addition resulted in an increase of N_L_ (+14% and +8% in N and O_3_N experimental sets, respectively); however, because of high variability in the data, no significant nitrogen effect (*p >* 0.05) was detected. However, C_L_ significantly increased in the evergreen species.

O_3_ caused the reduction in PNUE, because of slight, but not significant (*p* > 0.05), reduction of P_Nmax_ and N_L_, and an increase in C_L_ concentration. No interaction was detected. LMA decreased in *Q*. *ilex* after nitrogen addition, because leaf area increased (data not shown), influencing in the same direction as the LMA of the O_3_N experimental set.

### Antioxidant enzyme activities

**Fig 5:**
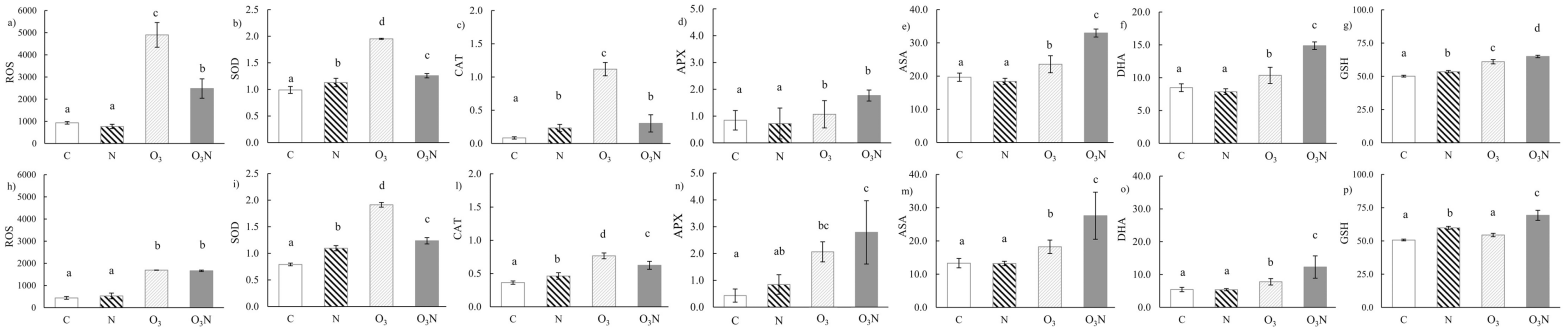
The outputs of biochemical analysis. They are shown for each treatment at the end of the experimental period for *F*. *ornus* (upper panel, from a to g) and *Q*. *ilex* (below panel, from h to p). Reactive oxygen species (ROS, %); superoxide dismutase, (SOD, inhibition rate %); catalase (CAT, U mg^-1^ of protein); ascorbate peroxidase (APX, U mg^-1^ of protein); total concentration of ascorbic acid (ASA, mg g^-1^); oxidised ascorbic acid (DHA, mg g^-1^); and glutathione (GSH, mg g^-1^). Data are means ± standard deviation (n = 5), and bars not accompanied by the same letter are significantly different at *p* < 0.05, by using post hoc Student–Neuman–Keuls test.

In both species, nitrogen addition increased SOD, CAT, and GSH activities, the key enzymatic components of the first antioxidant defense mechanisms involved in O_2_^-^ and H_2_O_2_ scavenging (Fig 5).

In *F*. *ornus*, the ROS amount increased significantly under O_3_ exposure; however, compared to that in C, their concentration in the O_3_ experimental set was higher than that in the O_3_N experimental set. Accordingly, the activity of the first line of ROS scavengers such as SOD and CAT were higher in O_3_ than in the O_3_N experimental set, but the antioxidants involved in the conversion of H_2_O_2_ to O_2_ or H_2_O (i.e. APX, ASA, DHA, and GSH) were upregulated in the O_3_N plants. Conversely, in *Q*. *ilex*, even if ROS were produced to the same extent as in O_3_ and O_3_N plants, SOD and CAT were lower in O_3_N than in O_3_. All the antioxidants related to ascorbate-glutathione cycle were higher in O_3_N than in O_3_ plants.

## Discussion

The impacts of atmospheric nitrogen deposition and O_3_ on Mediterranean forests have been of increasing concern, and experimental data are needed to elucidate the mechanisms of action, or identify specific functional traits affected by interacting stress factors. Thus, the present study aimed to measure the effects of realistic exposure of nitrogen and O_3_ on a broad range of traits of two Mediterranean species with different leaf habits, to characterize their response. The study was performed under controlled condition in a medium-term experiment, in order to determine the traits that are first affected by nitrogen and how the potential nitrogen effects can influence the response to O_3_. Moreover, to our knowledge, this is the first study to compare *F*. *ornus* and *Q*. *ilex* directly after O_3_ exposure, with important implication for assessing the risk for these co-occurring species.

### Response patterns to nitrogen deposition

An overview of available literature highlights that the results for the effects of nitrogen deposition at the leaf and plant levels are contradictory [37,66]. The numerous processes involved in nitrogen assimilation and metabolism might lead to high variability in the assayed data. In our experiment, after an acclimation period to nitrogen, when the cumulative dose was roughly equivalent to 14 kg N ha yr^-1^, which was within the threshold load considered as critical for Mediterranean vegetation, nitrogen concentration at the leaf level did not increase in both species, and no adverse effect was detected on photosynthetic traits (data not shown). When nitrogen exposure exceeded this level, between 14 and 17 kg N ha yr^-1^ (DOF 1), assimilation rate measured under steady state conditions was adversely affected in *F*. *ornus* possibly because of the nitrogen-induced decrease in stomatal conductance, as documented in several species [67,68]. Indeed nitrogen can affect g_s_ by changing the hydraulic conductivity [40], or by increasing nitric oxide (NO) emission as a side-reaction of the nitrate assimilation process [69]. In fact, NO is involved in the ABA-induced stomatal closure process [68,70]. Since the first mechanism was observed in long-lasting fertilisation experiments (from 2 to 5 years of nitrogen addition), we argued that, in our study, the nitrogen effect on g_s_ could be mediated by NO signalling.

Interestingly, in both species, P_Nmax_ and J_max_ increased because of nitrogen, whereas V_cmax_ did not change, namely, the electron transport driving RuBP regeneration was more affected than carboxylation. This result also explains why the assimilation rate measured under steady state condition, i.e. in the Rubisco-limited phase [59], did not show variations. Moreover, this evidence and the Chl *a* fluorescence measurement, indicate that, in *Q*. *ilex*, a higher fraction of nitrogen was allocated to components related to biochemical phase of assimilation process than to light-harvesting elements [71]. This hypothesis is not completely applicable to *F*. *ornus*. In fact, in *Q*. *ilex*, nitrogen significantly affects only the parameters related to the functionality of the end acceptors (i.e. IP-phase and PI_tot_); in contrast in *F*. *ornus* primary photochemistry (φ_Po_ and J-phase) was also enhanced by nitrogen, confirming that the partitioning pattern can differ depending on leaf habit [47].

In agreement with ecophysiological measurements, at the end of the experimental period, the concentration of nitrogen on mass basis increased to a different extent between the species. In *F*. *ornus*, nitrogen addition resulted in 25% higher N_L_ relative to that in the control (*p* = 0.058), whereas in *Q*. *ilex* the variation was less pronounced (+ 14%). Notably in both species PNUE did not change because of nitrogen addition. PNUE is controlled by physiological (assimilation rate, Rubisco activity, and nitrogen concentration on area basis) and structural traits (LMA, leaf thickness) [72], which can change in opposite ways and explain the lack of difference among treatments. In *F*. *ornus*, nitrogen treatment decreased both leaf area and dry mass, resulting in no LMA variation. However, in *Q*. *ilex*, the LMA decreased because of nitrogen, since the leaf area increased; thus, the evergreen species did not invest resources in the cell wall to increase leaf toughness, as reported in other studies [73,74].

These effects of nitrogen supply on leaf structural traits could influence the leaf nitrogen concentration when it is considered on area-basis [28,75]. In *F*. *ornus*, area-based leaf nitrogen was lower relative to the mass-based value (data not shown), whereas the two values remained similar in *Q*. *ilex*.

### Response patterns to ozone

Ozone effect was assessed using a wide range of traits to allow defining thoroughly the differences in the response patterns between the species. Plant sensitivity to O_3_ cannot be identified based on the extent of leaf injury alone, because impairments to photosystem functionality and photochemistry occur before the appearance of visible injury [54,76]. In our experiment, although we adopted a realistic O_3_ exposure (80 ppb h, AOT40 2458), *F*. *ornus* seemed to be sensitive to this pollutant because it did not trigger an active physiological response to O_3_, such as avoidance mechanisms, activating instead an incoming injury. The gas exchange reduction occurred from the first day of fumigation (-55% and -33% for g_s_ and P_N_, respectively), remaining around this order of magnitude for the entire fumigation period. Furthermore, in this species the P_N_ reduction was not merely owing to stomatal limitation, since the P_N_/Ci response curves highlighted a decrease of P_Nmax_ and of both carboxylation efficiency (V_cmax_) and maximum electron transport rate driving RuBP regeneration (J_max_). The reduction of nitrogen concentration can also be a good indicator of O_3_ injury, helping to define the scale of tolerance between species [23]. In *F*. *ornus*, O_3_ exposure accelerated the processes related to senescence as shown by the decrease of leaf nitrogen and dry matter i.e. decrease of LMA. Although the results from controlled conditions cannot be extended to natural ecosystems, the sensitivity found in this experiment should be considered for risk assessment of tree species in a Mediterranean climate. The response pattern of *Q*. *ilex* to O_3_ can be attributed to an avoidance mechanism, as shown by traits related to photochemistry, photosystems functionality or structural traits. The reduction of P_N_ in O_3_-treated plants relative to controls was less pronounced in *Q*. *ilex* than in *F*. *ornus*, starting on DOF 4, and was related to stomatal limitation more than to biochemical impairments. Indeed, the parameters derived from P_N_/Ci curves did not indicate any detrimental effect on Rubisco activity because of O_3_, whereas the analysis of photosystem functionality highlighted a down-regulation mechanisms (i.e. reduction of end-acceptors activity). The differences in the response strategies implemented by the two species could be strictly associated with a different antioxidant potential [11]. Indeed, the inherent amount of CAT, responsible for the removal of H_2_O_2_ [77], was higher in *Q*. *ilex*. Moreover, the components involved in the ascorbate-glutathione cycle (APX, ASA, DHA, and GSH) showed high concentration or activity in *Q*. *ilex* relative to that in *F*. *ornus*, but these could be related to the higher stomatal conductance (that is, high O_3_ fluxes) in the former species. Furthermore, in both the species, the finding that, in O_3_-treated plants, the increase in DHA/ASA ratio was lower than the increases in APX activity, suggestings that recycling of DHA to ASA was not compromised by O_3_.

### Response patterns to the interaction between nitrogen deposition and ozone

Information on the interactive effects of nitrogen deposition and ozone pollution on vegetation is still scarce [34]. Many of the studies on nitrogen and O_3_ have focused on the changes in community structure or species abundance in grasslands ecosystems [30,31,78]. However, few studies have determined the consequences of nitrogen and O_3_ interaction on tree species [24,38,43]. The results highlighted by previous studies suggested that the interactive effects could be dynamic, changing throughout the growing season, and the effects on key ecophysiological parameters such as P_N_ and g_s_ can remarkably change depending on the concentration of nitrogen and O_3_ exposure. In the present study, the interaction followed different patterns in the two species, confirming our hypothesis that leaf habit plays a crucial role in determining the way of interaction between the two factors. In particular, in *F*. *ornus*, the interaction was detectable on several traits indicating that nitrogen addition can ameliorate the detrimental effects owing to O_3_. Nitrogen had a positive effect on the processes related to photochemistry, resulting in enhanced carbon assimilation rate in the O_3_N experimental set relative to that in the O_3_ experimental set. The mechanisms involved in this type of response could be associated with the investment of available nitrogen to proteins that play a crucial role in enhancing the photosynthetic activity [79]. We argue that nitrogen was partially allocated to light-harvesting components, increasing the capacity to manage the energy flow through the photosystems even if stomatal closure occurs [57]. The positive effect of nitrogen on the functionality of plants treated with O_3_ could also be attributed to the upregulation of antioxidant response to O_3_ implemented by nitrogen addition [80]. In particular, in O_3_N plants, the APX activity and ASA and DHA were higher compared to that after treatment with O_3_ alone. Moreover, in *F*. *ornus*, the O_3_N experimental set led to lower ROS production relative to that in O_3_ plants, that is, lower exposure to oxidative stress, even if the O_3_ fluxes remained almost the same. On average, during the entire experimental period, g_s_ was about 96.9 ± 21.2 and 92.5 ± 12.3 in O_3_ and O_3_N, respectively. This could be because of NO synthesis. Plants can emit NO under a series of stresses [81], in particular under ozone exposure [7], and several studies revealed that high nitrogen availability can promote NO production [82]. NO is involved in triggering antioxidant response and can react directly with free radicals such as H_2_O_2_ and O_2_^−^, thereby decreasing their concentrations. We argue that, in the presence of high nitrogen availability, *F*. *ornus* can cope with incoming oxidative stress via the production of NO, which owing to the rapid synthesis and prompt availability, allows prompt scavenging of free radicals and concomitantly increases the photosynthesis rate [83]. As shown by Velikova et al. [78], higher NO is emitted in isoprene-inhibited leaves; therefore NO synthesis should be favoured in plants that do not emit VOCs, such as *F*. *ornus*. In *Q*. *ilex*, a strong monoterpene emitter species [84], the response to oxidative compounds could be attributed to VOCs [85] rather than to NO. Most importantly, in both species, nitrogen led to the increase in the constitutive amount of antioxidant enzymes such as SOD and CAT, but did not affect their activity (GST did not change, data not shown).

Unlike in *F*. *ornus*, in *Q*. *ilex*, the interaction between nitrogen and O_3_ was weak and appeared only on DOF 1, when nitrogen seemed to aggravate the stomatal limitation owing to O_3_. The g_s_ of O_3_N plants recovered at the following sampling times to the level in the O_3_ experimental set.

Photosystem functionality was not affected by the interaction, and Rubisco-related parameters did not show any nitrogen effect (positive or negative) when plants were exposed to O_3_. However, interestingly, enzymes such as SOD and CAT, which play a crucial role in determining a suitable level of ROS in different cell compartments [77], were lower in O_3_N plants than in O_3_ plants, although the ROS production did not differ between the treatments. This evidence suggests that, in *Q*. *ilex*, nitrogen addition could enhance secondary metabolism and promote the production of VOCs that can directly quench O_3_ without activating an enzymatic antioxidant response. Conversely, similar to that in *F*. *ornus*, nitrogen upregulated the activity of the ascorbate-glutathione cycle in *Q*. *ilex*, indicating that nitrogen deposition can largely protect against oxidative stressors and to multi-stress condition experienced by Mediterranean vegetation.

## Conclusion

In both species, photosynthetic traits such as photosystem functionality, maximum assimilation and maximum electron transport rate were enhanced by nitrogen at the end of the experiment, when 20 kg N ha^-1^ y^-1^ had been reached. Moreover, in both species, nitrogen enhanced the constitutive level of antioxidant activity, thus with a potential ameliorative effect on O_3_-related impacts. In *F*. *ornus* nitrogen was allocated to the light harvesting components as shown by chlorophyll *a* fluorescence measurements. The results suggest that *F*. *ornus* is an O_3_-sensitive species, as shown by biochemical limitation to photosynthesis and the acceleration of leaf senescence-related processes. In *F*. *ornus*, nitrogen ameliorated the detrimental effects that O_3_ had on the photosynthetic processes. The interaction between nitrogen and O_3_ had different mechanisms of action in the two species. In the deciduous species *F*. *ornus*, the lower ROS production in the interaction experimental set (O_3_N) might be related to the enhanced nitrogen oxide production, whereas, in *Q*. *ilex*, nitrogen might have upregulated the secondary metabolism, promoting high VOCs production. This hypothesis is based on the fact that, although no difference was noted in ROS production between O_3_ and O_3_N plants in this species, the activity of the first-level scavenging enzymes such SOD or CAT was lower in the interaction experimental set. These results indicate that nitrogen deposition could counteract the detrimental effect of O_3_, thus suggesting that nitrogen is an important factor for assessing the critical level of O_3_ for Mediterranean vegetation.
